# Carotenoid Profile of Tomato Sauces: Effect of Cooking Time and Content of Extra Virgin Olive Oil

**DOI:** 10.3390/ijms16059588

**Published:** 2015-04-28

**Authors:** Anna Vallverdú-Queralt, Jorge Regueiro, José Fernando Rinaldi de Alvarenga, Xavier Torrado, Rosa M. Lamuela-Raventos

**Affiliations:** 1 INRA, UMR1083 Sciences pour l’œnologie, 2 Place Pierre Viala, Montpellier Cedex 34060, France; E-Mail: avallverdu@ub.edu; 2 CIBER Fisiopatología de la Obesidad y la Nutrición (CIBEROBN), Instituto de Salud Carlos III, 28029 Madrid, Spain; 3 Nutrition and Bromatology Group, Analytical and Food Chemistry Department, Faculty of Food Science and Technology, Ourense Campus, University of Vigo, E-32004 Ourense, Spain; E-Mail: jorge.regueiro@uvigo.es; 4 Department of Food Science and Nutrition, School of Pharmaceutical Science, São Paulo State University—UNESP, Rod. Araraquara-Jaú Km 1, 14801-902 Araraquara, SP–CEP 14801-902 São Paulo, Brazil; E-Mail: zehfernando@gmail.com; 5 Nutrition and Food Science Department, XaRTA, INSA. Pharmacy School, University of Barcelona, Av. Joan XXIII s/n, 08028 Barcelona, Spain; E-Mail: torrado@ub.edu

**Keywords:** LC-ESI-MS/MS, LC-UV, C30 column, β-carotene, α-carotene, lycopene, antioxidant capacity

## Abstract

The consumption of carotenoid-rich vegetables such as tomatoes and tomato sauces is associated with reduced risk of several chronic diseases. The predominant carotenoids in tomato products are in the (*all*-*E*) configuration, but (*Z*) isomers can be formed during thermal processing. The effect of cooking time (15, 30, 45 and 60 min) and the addition of extra virgin olive oil (5% and 10%) on the carotenoid extractability of tomato sauces was monitored using liquid chromatography-tandem mass spectrometry (LC-ESI-MS/MS) and LC-ultraviolet detection (LC-UV). The thermal treatment and the addition of extra virgin olive oil increased the levels of antioxidant activity, total carotenoids, *Z*-lycopene isomers, α-carotene and β-carotene. These results are of particular nutritional benefit since higher lycopene intake has been associated with a reduced risk of lethal prostate and a reduction of prostate-specific antigen (PSA) levels. Moreover, β-carotene has been reported to suppress the up-regulation of heme oxygenase-1 gene expression in a dose dependent manner and to suppress UVA-induced *HO*-*1* gene expression in cultured FEK4.

## 1. Introduction

The traditional Mediterranean diet is characterized by a high intake of olive oil, fruit, nuts, vegetables, and cereals [1,2]. Tomato and tomato sauces are typical components of the Mediterranean diet and are of great interest because of their high content of bioactive compounds, including carotenoids, polyphenols, and vitamin C. Regular consumption of tomato products has been related to a decrease in the incidence of chronic degenerative diseases [3,4]. Some carotenoids, such as β-carotene and (*all-E*)-α-carotene, may exhibit provitamin A activity [5]. Studies have demonstrated that consumption of lycopene decreases the risk of degenerative diseases, for example, certain kinds of cancer and cardiovascular diseases [6]. Another major characteristic of the Mediterranean diet is a high consumption of olive oil, which is associated with numerous health benefits [7,8]. Oil added to tomato sauce has been reported to improve the accessibility and extractability of bioactive compounds in tomato [9]. Gärtner *et al.* [10] reported that with a constant content of fat and other ingredients, lycopene bioavailability from tomato paste was significantly higher than from fresh tomatoes [10].

Thermal treatments are the main cause of the depletion of natural antioxidants in food. Browning and oxidation reactions are responsible for the degradation of naturally occurring antioxidants during the processing of tomato products [11,12]. The highly unsaturated nature of carotenoids makes them susceptible to isomerization, oxidation and breakdown of the carotenoid molecule during thermal processes, especially under severe processing conditions. The oxidation products formed are a mixture of epoxides, apocarotenals and hydroxy compounds [13]. During thermal treatments, (*Z*)*-*isomers are formed. Studies have proven that >50% of the carotenoids present in the human body are in the (*Z*) isomeric configuration, suggesting that this is the most bioavailable form [14].

The effects of industrial processing on the lycopene content of tomatoes have been extensively studied. However, little is known about the impact of cooking time or added extra virgin olive when tomato products are prepared at home. Thus, the aim of this study was to examine how the duration of cooking (15, 30, 45 and 60 min) and the addition of extra virgin olive oil (5% and 10%) affected the carotenoid content of tomato sauces.

## 2. Results and Discussion

While some studies have reported a loss of lycopene in tomato-based foods undergoing thermal processing (bleaching, retorting and freezing) [15], others have shown that processing tomatoes may increase the levels of some bound antioxidants [9,16,17]. As carotenoids are widely present in food, the assessment of their stability in food systems is of major importance.

**Figure 1:**
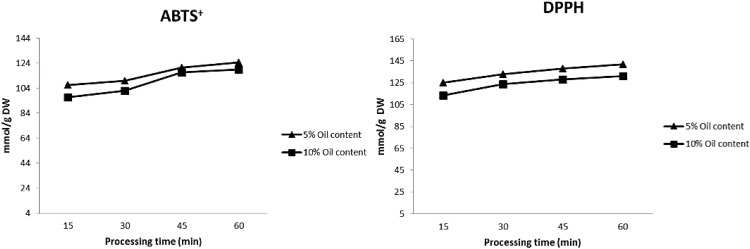
Changes in antioxidant capacity during processing time.

Analysis of the tomato sauces during the home-cooking processes showed that the antioxidant level measured by the ABTS^+^ assay increased from 106.68 mmol/g DW at 15 min to 124.57 mmol TE/g DW at 60 min or from 96.91 mmol TE/g DW at 15 min to 119.08 mmol TE/g DW at 60 min in sauces containing 5% or 10% extra virgin olive oil, respectively (Figure 1). The same pattern of antioxidant capacity was observed in the DPPH assay. Food processing may improve carotenoid bioavailability by breaking down cell walls, which weakens the bonding forces between carotenoids and the plant tissue matrix. Piga *et al.* [18] reported an increase in the DPPH content of mandarin juices over time, and attributed this increase in antioxidant capacity to the formation of Maillard’s reaction products [18]. The food matrix in which the bioactive compounds are contained also plays a crucial role in determining their accessibility and extractability from food [9].

**Figure 2:**
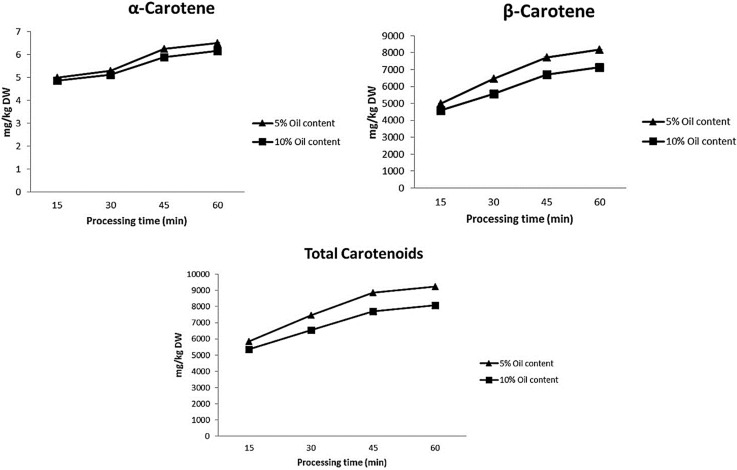
Changes in total carotenoids, α-carotene and β-carotene during processing time.

**Figure 3:**
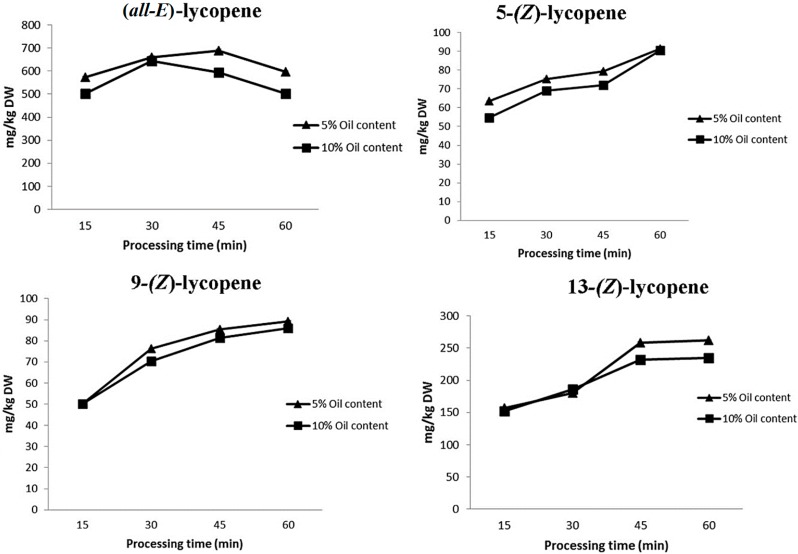
Changes in (*all-E*)-lycopene and 5-(*Z*), 9-(*Z*) and 13-(*Z*)-lycopene during processing time.

Figure 2 and Figure 3 show the evolution of the carotenoid content in the studied tomato sauces during the cooking process. The concentration after 15 min of processing of (*all-E*)-lycopene was 573.76 ± 45.83 mg/kg·DW, whereas those of (*all-E*)-α-carotene and (*all-E*)-β-carotene were 4.99 ± 0.13 and 4998.5 ± 268.95 mg/kg DW, respectively (Figure 2 and Figure 3). Between 15 and 30 min of cooking, (*all-E*)-lycopene increased from 573.76 to 659.27 mg/kg·DW in tomato sauces containing 5% olive oil and from 503.37 to 643.46 mg/kg DW in those with 10% olive oil. Between 30 and 60 min, the (*all-E*)-lycopene content decreased from 659.27 to 597.11 mg/kg DW in tomato sauces containing 5% olive oil, with a similar pattern being observed in sauces containing 10% olive oil. In contrast, (*all-E*)-α-carotene and (*all-E*)-β-carotene increased over the cooking period (15 to 60 min) from 4.99 to 6.50 mg/kg DW and from 4998.5 to 8185.12 mg/kg DW, respectively. No statistically significant differences were found between samples containing 5% or 10% olive oil at the same time of processing. Gärtner *et al.* [10] reported that tomato juice cooked in oil medium resulted in a two- to three-fold increase in carotenoid serum concentrations one day after ingestion, while no rise was found after an equivalent consumption of unprocessed tomato juice [10].

Our results are in line with those reported by Lin and Chen [19], who attributed the lower stability of (*all-E*)-lycopene to its coplanar structure with 11 conjugated double bonds, which results in a higher reactivity in comparison with (*all-E*)-β-carotene [19]. In contrast, tomato processing may activate enzymes involved in the synthesis of β- and α-carotene. The carotenoid biosynthetic pathway in plants has two main branches after lycopene, distinguished by different cyclic end-groups. Two β rings lead to the β,β branch (β-carotene and its derivatives) through β-carotene cyclase, while one beta and one epsilon ring define the β,ε branch (α-carotene and its derivatives) through ε- and β-carotene cyclase. Thermal processing may activate ε*-* and β*-*carotene cyclase and, thus stimulate α- and β-carotene production.

The (*Z*)-isomers of lycopene during processing were also studied since these compounds are of particular nutritional benefit due to their ready absorption in the human intestine [20]. We tentatively identified three isomers in tomato sauces: 5-, 9- and 13-(*Z*)-lycopene (Figure 3). This lycopene isomer profile is in accordance with that reported by other authors in different tomato products, in which (*all-E*)-lycopene represents the most abundant lycopene isomer, varying from 35% to 96% of total lycopene, and 5-, 9-, 13- and 15-(*Z*)-lycopene are the main (*Z*)-isomers detected [21,22]. We observed higher levels of (*Z*)-lycopene in sauces containing less virgin olive oil. For instance, at 45 min of cooking the concentration of 5-(*Z*)-lycopene was 79.35 mg/kg DW with 5% olive oil as opposed to 72.06 mg/kg DW with 10% olive oil.

It has been shown that during the cooking process lycopene undergoes (*all-E*)/(*Z*)-isomerization, leading to an increase in the proportion of (*Z*)-isomers [19]. As mentioned above, (*all-E*)-lycopene underwent a loss of 9% during the preparation of tomato sauces, in contrast with (*Z*)-lycopene isomers, which increased slightly, by 31%, 18% and 15% in the case of 13-, 5- and 9-(*Z*)-lycopene, respectively (Figure 3). These results are in agreement with those reported by Lin and Chen [19], who observed the formation of (*Z*)-lycopene isomers in tomato juice [19]. This could be explained by the isomerization phenomenon: (*all-E*)-lycopene can be converted to 13-(*Z*)-lycopene, which can then undergo subsequent conversion into other (*Z*)-isomers [23]. Similar lycopene degradation patterns have been described by Giovanelli and Paradiso [24] during the processing and storage of tomato paste. The biosynthetic enzymes involved in carotenoid biosynthesis in plants are encoded by nuclear genes, and precursor proteins are post-translationally imported into plastids, where carotenoid biosynthesis takes place. Disruption of tissue by wounding could promote the transcription of the genes or the transport of the mRNA related to the synthesis of carotenoids [25]. The production of (*Z*)-lycopene isomers due to thermal treatments could be explained by the activation of enzymes such as phytoene synthase and ζ-carotene desaturase (ZDS). Phytoene undergoes a set of desaturation reactions, each of which creates a new double bond and extends the chromophore by two conjugated double bonds; the end product is lycopene [26]. Controversially, García Alonso *et al.* [27] found that the total lycopene content remained quite stable throughout the storage trial and varied from 99 to 120 mg/kg in tomato juice packaged in Tetra Paks and from 96 to 115 mg/kg in samples stored in glass bottles. For both types of samples, no clear temperature dependency was revealed regarding the rate of total lycopene loss, which was affected only by storage time [27].

The thermal treatment increased the levels of antioxidant activity and total carotenoids by 15% and 30%, respectively. As mentioned above, 13-, 5- and 9-(*Z*)-lycopene increased by 31%, 18% and 15%, respectively. The (*Z*)-isomers of lycopene during processing are of particular nutritional benefit due to their ready absorption in the human intestine [10]. Higher lycopene intake is associated with a reduced risk of lethal prostate [28] and an increase of levels of lycopene in sauce may help to reduce PSA [29]. Moreover, (*all-E*)-α-carotene and (*all-E*)-β-carotene increased over the cooking period by 25% and 40%, respectively. Dietary β-carotene has been reported to decrease (distal) colon cancer [30] and moreover, to suppress the up-regulation of heme oxygenase-1 gene expression in a dose dependent manner and to suppress UVA-induced *HO*-*1* gene expression in cultured FEK4 [31]. Moreover, it seems that these compounds may act synergically increasing their anticancer activity [32].

## 3. Experimental Section

### 3.1. Standards

All samples and standards were handled without exposure to light. β-carotene, α-carotene, lycopene, methyl tert-butyl ether (MTBE), ABTS: 2,2'azino-bis(3-ethylbenzothiazoline-6-sulfonic acid), Trolox: (±)-6-hydroxy-2,5,7,8-tetramethylchromane-2-carboxylic acid 97% and manganese dioxide and hexane were purchased from Sigma^®^ (St. Louis, MO, USA); ethanol and methanol HPLC grade were obtained from Scharlau (Barcelona, Spain); DPPH: 2,2-diphenyl-1-picrylhydrazyl from Extrasynthèse (Genay, France); and ultrapure water (Milli-Q) from Millipore (Bedford, MA, USA).

### 3.2. Tomato Sauce Material

A commercial tomato (*Licopersicon esculentum* Mill. c.v. Daniella), suitable for tomato sauce making, was used for the study. Virgin olive oil was kindly furnished by Manuel Heredia Halcón (Cortijo De Suerte Alta, Albendin-Baena-Cordoba). The tomato sauces were prepared at Torribera Campus, University of Barcelona (UB, Barcelona, Spain), following a conventional home cooking method. Fruits were washed, chopped in a breaker unit, weighed and crushed with a Thermomix^®^. Two variables were evaluated: virgin olive oil addition at two different concentrations and the cooking time. The olive oil (5% and 10% of oil, *w*/*w*) was added to the chopped tomatoes, and the mixture was cooked at 95–96 °C for 15, 30, 45 or 60 min. The tomato sauces were aliquoted and stored in vacuum bags at −20 °C until the day of the analysis. A 4 × 2 mixed-level factorial design was performed, involving a total of 8 randomized runs. For the reproducibility, each process was evaluated three times. The carotenoids profile of tomato sauce control without addition virgin olive oil are reported in Table 1.

**Table 1:** The carotenoids profile of tomato sauce control without addition virgin olive oil.

Compounds	Tomato Sauce Control (mg/kg DW)
**α-carotene**	4.3 ± 0.3
**β-carotene**	3266.9 ± 53.1
**(*all-E*)*-*Lycopene**	178.9 ± 10.1
**5-(*Z*)-Lycopene**	20.3 ± 1.1
**9-(*Z*)-Lycopene**	26.2 ± 0.9
**13-(*Z*)-Lycopene**	33.6 ± 2.7
**Total Carotenoids**	3530.1

### 3.3. Extraction and Analysis of Carotenoids

#### 3.3.1. Extraction of Carotenoid Compounds

The extraction of carotenoids was carried out in darkness using dry ice in order to minimize autoxidation and (*Z*)-(*all-E*) isomerization, and to avoid exposure to light, oxygen and high temperatures.

Tomato sauces (0.5 gram) were weighed and homogenized with 5 mL ethanol:n-hexane (4:3, *v*/*v*) following a procedure described in the literature [33]; they were then sonicated for 5 min and centrifuged (4000 rpm at 4 °C) for 15 min. The supernatant was transferred into a flask and extraction was repeated. The supernatants were combined and evaporated under nitrogen flow; finally, the residue was reconstituted with MTBE up to 1 mL and filtered through a 25 mm, 0.45 µm PTFE filter (Waters, Mildford, MA, USA).

#### 3.3.2. Analysis of Carotenoid Compounds

Chromatographic analysis was performed using the HPLC system previously described by Vallverdu-Queralt *et al.* [28]. The analytes were separated on a reversed-phase C30 column YMC30 (250 × 4.6 mm, 5 μm) from YMC (Dinslaken, Germany) and kept at 20 °C. The injection volume was 20 µL and flow rate 1 mL·min^−1^. The mobile phase consisted of two different solvent mixtures: A—water: MTBE:methanol (4:26:70, *v*/*v*/*v*)—and B, with the same solvents but other proportions (4:90:6, *v*/*v*/*v*). The linear gradient was 26% B to 90% B in 23 min. The column was equilibrated for 10 min prior to each analysis. MTBE was used as a modifier to facilitate the elution of lycopene, which is strongly retained in a methanol environment [34].

### 3.4. Identification and Quantification of Carotenoids

#### 3.4.1. Diode Array Detector

Commercially available carotenoid standards ((*all-E*)*-*α*-*carotene, (*all-E*)*-*β-carotene and (*all-E*)-lycopene) were used to identify analytes by retention times and UV-VIS spectra. The LC-DAD chromatograms were acquired by selecting the 450 nm wavelength; in addition, the UV-VIS spectra were recorded in the range of 350–550 nm for the tentative identification of carotenoids and their geometrical isomers ((*Z*)-lycopene isomers), on the basis of the retention times and absorption spectrum characteristics described in the literature [21,34].

#### 3.4.2. Mass Spectrometry

The API 3000 (PE Sciex, Concord, ON, Canada) triple quadrupole mass spectrometer in positive-ion mode was used to obtain MS/MS data for carotenoid analysis. Turbo Ionspray source settings were the same as previously described by our group [33]. Post-column addition of a solution of LiCl (500 mg·L^−1^) was performed using an isocratic pump Agilent 1100 Series (Agilent Technologies, Palo Alto, CA, USA) at a flow rate of 100 µL·min^−1^ via a zero-volume mixing T-piece.

Carotenoids were quantified with respect to their corresponding standard according to the internal standard method using *trans*-β-apo-8'-carotenal. When standards were not available, as in the case of (*Z*) isomers of lycopene, they were quantified on the basis of the peak area of the (*all-E*)-lycopene standard. Results are expressed as mg/kg dry weight (DW).

### 3.5. Antioxidant Capacity

The antioxidant capacity of tomato sauces was measured using ABTS^+^ and DPPH assays reported in the literature [33].

#### 3.5.1. ABTS^+^ Assay

One mM Trolox (antioxidant standard) was prepared in methanol once a week. Working standards were prepared daily by diluting 1 mM Trolox with methanol. 

An ABTS^+^ radical cation was prepared by passing a 5 mM stock solution of ABTS (in methanol) through manganese dioxide powder. Excess manganese dioxide was filtered through a 13 mm 0.45 µm filter PTFE (Waters). Before analysis, the solution was diluted in methanol pH 7.4 to give an absorbance at 734 nm of 1.0 ± 0.1, and pre-incubated in ice. Then, 245 µL of ABTS^+^ solution was added to 5 µL of Trolox or to tomato extracts and the solutions were stirred for 30 s. The absorbance was recorded continuously every 30 s with a UV/VIS spectrophotometer Thermo Multiskan Spectrum from Thermo Fischer Scientific (San Jose, CA, USA) for 1 h and methanol blanks were run in each assay.

The working range for Trolox (final concentration 0–750 µmol/L) was based on triplicate determinations and consisted of plotting the absorbance as a percentage of the absorbance of the uninhibited radical cation (blank). The activities of the tomato extracts were assessed at four different concentrations, which were within the range of the dose-response curve. Each sample was analyzed in triplicate at each concentration. Results were expressed as mmol Trolox equivalent (TE)/g DW.

#### 3.5.2. DPPH Assay

The antioxidant capacity was also studied through the evaluation of the free radical-scavenging effect on the DPPH radical. Solutions of known Trolox were used for calibration. 5 µL of tomato extracts or Trolox were mixed with 250 µL of methanolic DPPH (0.025 g/L). The homogenate was shaken vigorously and kept in darkness for 30 min. Absorption of the samples was measured on a UV/VIS Thermo Multiskan Spectrum spectrophotometer at 515 nm. The percentage of inhibition of the DPPH activity was calculated and plotted as a function of the Trolox concentration for the standard reference data. The final DPPH values were calculated using a regression equation between the Trolox concentration and the percentage of DPPH inhibition and results were expressed as mmol TE/100 g DW.

### 3.6. Statistical Treatments

Treatments of tomato sauces were carried out in triplicate and each replicate was analyzed three times. Significance of the results and statistical differences were analyzed using Statgraphics plus v. 5.1 software (Manugistics, Rockville, MA, USA). Data were analyzed by multifactor analysis of variance and a Duncan multiple range test was applied to determine differences among means, with a significance level of *p* = 0.05.

## 4. Conclusions

In this work, the effects of cooking time (15, 30, 45 and 60 min) and the amount of added virgin olive oil (5% and 10%) on the carotenoid profile of tomato sauces were studied using LC-ESI-MS/MS and LC-UV. During the thermal treatment, levels of antioxidant activity, total carotenoids, α-carotene, β-carotene and (*Z*)-lycopene increased. This information may be used for industries to develop new treatments so as to obtain products with high carotenoid content and higher physiological effects. This increase in carotenoids and antioxidant capacity may reduce lipid peroxidation, increase HDL and decrease the incidence of chronic degenerative diseases, such as cancer and cardiovascular diseases.
